# Perception of health, health behaviours and the use of prophylactic examinations in postmenopausal women

**DOI:** 10.1186/s12905-020-00931-9

**Published:** 2020-04-09

**Authors:** Anna B. Pilewska-Kozak, Klaudia Pałucka, Celina Łepecka-Klusek, Grażyna Stadnicka, Krzysztof Jurek, Beata B. Dobrowolska

**Affiliations:** 1 Chair and Department of Gynecology and Gynecological Endocrinology, Faculty of Health Sciences, Medical University of Lublin, Aleje Racławickie 23, 20-049 Lublin, Poland; 2 Chair and Department of Development in Midwifery, Faculty of Health Sciences, Medical University of Lublin, Lublin, Poland; 3 Faculty of Social Sciences, Institute of Sociology, John Paul II Catholic University in Lublin, Lublin, Poland; 4 Chair of Development in Nursing, Faculty of Health Sciences, Medical University of Lublin, Lublin, Poland

**Keywords:** Health behaviours, Postmenopause, Prophylactic actions, Women

## Abstract

**Background:**

Pro-health behaviours aim at disease prevention, recovery from an illness and maintenance of good health in a physical, mental and social sphere. The study had two main objectives: (a) to analyse health behaviours of postmenopausal women and their understanding of the notion of health, and (b) to analyse the relationship between individual categories of health behaviours and prophylactic activities undertaken by postmenopausal women.

**Methods:**

A cross-sectional study was conducted among a convenience sample of 510 postmenopausal women. Three study instruments were used: an original questionnaire and two instruments designed by Juczyński: the List of Health Criteria and the Health Behaviour Inventory.

**Results:**

In the view of the respondents health was primarily synonymous with a feature, because the following three associations were given the highest priority when defining health: to be healthy means ‘have all body parts functioning well’ (M = 1.82), ‘do not experience any physical problems’ (M = 1.43) and ‘not be sick, only occasionally suffer from flu, cold or indigestion’. The score for health behaviours was average (M = 86.18). The highest score was achieved in the area of prophylactic behaviours. General indicator of health-related behaviours was higher in women who rated their health as very good (*p* < 0.05). Women whose general indicator of health-related behaviours was higher regularly performed prophylactic gynaecological examinations (*p* < 0.05).

**Conclusions:**

Results showed that women after menopause treat health mainly as a feature of their body and condition specific for this period of life. The analysis of postmenopausal women’s health behaviours and their perception of health helped to identify areas that require the focus of medical personnel in regard to health promotion and prophylaxis. The average general indicator of health-related behaviours is positive for this group of women as it shows that they care about their health, especially in terms of prophylaxis.

## Background

The main functioning paradigm of health is the holistic (sociomedical) model. It comprehensively follows the definition of health developed by the experts of World Health Organization (WHO). Health is a relative state where a person can function well physically, mentally, socially and spiritually and express a full range of unique abilities in the environment in which he or she lives [1, 2]. Understanding health in everyday life may differ from the accepted scientific concepts and may be different for different individuals. For many people the main indicators of being healthy are not experiencing symptoms of any disease and having the ability to play social roles [3, 4].

Health behaviours are all activities related to health and well-being, including activities that promote health (pro-health) and activities that contribute to the destruction of health (anti-health). Pro-health behaviours aim at disease prevention, recovery from an illness and maintenance of good health in physical, mental and social sphere. Anti-health behaviours, on the other hand, may lead to psychophysical disorders [5, 6]. Generally speaking, pro-health behaviours should be a priority choice for everyone throughout life.

Women’s health is exposed to many challenges, both during and after reproduction. During reproductive stage women may be affected by several reproductive diseases. One of the most prevalent is endometriosis, the symptoms of which significantly affect the psychological and social functioning of women [7, 8]. The postmenopausal stage is the time of many complex biological changes in the female body. The changes are related to the gradual declining of the endocrine function of the ovaries, which may trigger many psychophysical problems. At the same time the risk of metabolic diseases and cancer increases [9, 10]. Another inherent element of the postmenopausal period are the characteristic changes of psychosocial nature - in addition to health issues problems concerning family life and work start to emerge and may have a significant impact on the quality of life and health of postmenopausal women [10, 11]. Self-esteem diminishes and the perception of their own femininity is altered [10, 12]. Therefore, engaging in pro-health activities may significantly improve the condition of women in the postmenopausal period and reduce the intensity of experiencing climacteric syndrome symptoms [5, 13–16]. In order to maintain good health and well-being it is sometimes advisable to modify the lifestyle and certain health behaviours already in the period prior to menopause. The main aspects include a balanced diet, active recreation, avoidance of stressful situations as well as undergoing regular preventive examinations [5].

Analysis of the literature shows that there are many studies on women’s health behaviours during and after the menopause. Investigators focused on health-enhancing behaviours and onanti-health activities. They examined the impact of a single activity and of several interacting activities on women’s health [12, 16–20]. However, there are only few studies on understanding of the concept of health by women in the postmenopausal period, and there are not so many analyses of the whole spectrum of health behaviours undertaken by this group, which were measured by standardized questionnaires. Therefore, this study had two main objectives: (a) to analyse health behaviours of postmenopausal women and their understanding of the notion of health, and (b) to analyse the relationship between individual categories of health behaviours and prophylactic activities undertaken by women in this stage of life.

## Method

### Design and setting

A cross-sectional study was conducted among a convenience sample of 510 post-menopausal women. The material was collected in gynaecological outpatient clinics (gynaecological clinics providing services of gynaecologists and other specialists involved in gynaecological care and midwives) and in primary care clinics (clinics providing services of general practitioners, primary care physicians, nurses and midwives) in the city of Lublin (Poland) from January 2016 to July 2017. The study was reported according to the Strengthening the Reporting of Observational studies in Epidemiology (STROBE) checklist [21].

### Study sample

The size of the study group was calculated taking into account the estimated data. The assumption was to conduct the study on group of women who were in postmenopausal period and therefore could refer to their experiences concerning that condition when filling out the questionnaires. Since in Poland the age of women who are going through menopause varies between 40 and 58 years of age [21], this age span was taken into account, and a margin of 5–7 years was added to ensure that the respondents have reached the postmenopausal period at the time of the study (according to the definition of an early and late postmenopausal period) [22, 23]. It was therefore assumed that the study should be performed on the group of women aged 45–65 years of age. According to the Central Statistical Office, the number of women aged between 45 and 65 years of age constitutes 27% of the total population of women in Poland (5445,165) [24]. With such a population, the study group should comprise a minimum of 384 participants (confidence interval = 0.95; maximum error 5%). Bearing this in mind, 545 women were invited to take part in the study. The response was very good. Only 35 women refused to participate (6.4%), claiming that they did not have time to complete the questionnaire. The remaining group (*n* = 510) completed the questionnaires, providing all the required answers, which allowed them to be included in the analysis.

The inclusion criteria included:

- the time which has lapsed since the final menstrual period (FMP): 2–10 years (this information was obtained from the respondents when they were invited to participate in the study, and later confirmed in writing, when they provided answers in the survey questionnaire)
- written consent for participation in the study;
- good general health condition
  Women after surgical menopause, women with history of psychiatric disorders and those who were not able to complete the questionnaire by themselves were excluded from the study.

A consent to carry out the study was obtained from the directors of the clinics. Coordinating nurses/midwives (coordinators who manage the nursing personnel in the clinics where the study was conducted) were asked to assist with the study. All participants reported to the clinic for a medical check-up. When the coordinating personnel identified a woman in the postmenopausal period, she was invited to take part in the study and to complete the questionnaire.

### Ethical issues

The study was carried out in accordance with the protocol accepted by the Bioethics Committee at the Medical University of Lublin (No: 0254/292/2015) and in accordance with the ethical principles of the Declaration of Helsinki. Each woman was individually invited to take part in the study and written consent was obtained from the women who agreed to participate. It was emphasized that the participation in the study was anonymous and voluntary. Participants gave their consent by signing a special document containing a detailed description of the purpose and the outline of the study. The participants were invited to a dedicated room to ensure intimacy when completing the questionnaire. There was no time limit imposed for completing the questionnaire. All respondents were given the opportunity to ask questions at every stage of the study and were provided with comprehensive answers.

### Research instruments

1. Short form of a retrospective nature which aim was to collect sociodemographic data and the obstetric and gynaecological history of the participants. Respondents were also asked to provide information concerning their health self-assessment, their health behaviours and their attitudes towards prophylactic examinations. This short form was developed by authors.
2. The List of Health Criteria (LHC) developed by Juczyński consists of 24 items defining positive elements of the physical, mental and social dimensions of health. Health was defined as: the goal, the process, the condition, the feature and the result. The study allowed the possibility of supplementing the list with additional definitions of health. Respondents selected five criteria which in their opinion were important for health assessment and ordered them from the most important (5 points) to the least important (1 point). The criteria which were not selected were assigned 0 points. The points corresponding to the selected criteria were the basis for the interpretation of the results. The interpretation was based on the average values provided by the author [25]. Coefficient alpha reliability of this instrument is 0.68 [25].
3. Inventory of Health Behaviour (IHB) developed by Juczyński included 24 terms describing specific groups of behaviours related to health. The author determined the intensity of behaviours that positively affect health and also the intensity of the following four groups of health behaviours: healthy eating habits, prophylactic behaviours, positive mental attitude, and health practices. Respondents, using a five-point scale (1 –hardly ever, 2 - seldom, 3 - from time to time, 4 - often, 5 - almost always), indicated how often they performed the given health behaviour in the previous year. The results were then summed up. The index value ranged from 24 to 120 points -the higher the score the greater the frequency of a health behaviour. Next, using a special table, the general indicator was converted into sten scores. Sten scores of 7–10 were considered to be high, sten scores of 5–6 were average and 1–4 were perceived as low. A diagnostic key designed by Juczyński was used to add up the points separately for each group of health behaviours. The results for each individual group, obtained by dividing the total score by 6, were compared with the gender-standardized table [25]. The internal consistency of this instrument was measured with Cronbach’s alpha and equals 0.85 for the whole scale [25].

Instruments 2 and 3 were purchased from the Psychological Test Laboratory in Warsaw. They were selected because of their good psychometric properties. Additionally, the IHB instrument had already been tested on a group of menopausal women [25]. Another important criterion was the fact that both instruments are available in the respondents’ mother tongue and were standardized for the population of women and men (IHB) and different age groups and environments (LHC) [25].

### Statistical analysis

The statistical analysis of the collected study material was performed with the IBM SPSS Statistics software. The quantitative variables were described as arithmetic means with standard deviation, and the qualitative variables were expressed with the number of indicated response categories and percentage. Additionally, to verify the hypotheses, appropriate statistical procedures were applied. Comparison of two groups was performed with the Mann-Whitney U test. This test is used to verify the hypothesis concerning the irrelevance of differences between medians of the studied variable in two populations, assuming that the distributions of the variables are close. Comparison of more than two independent groups was performed with the Kruskal - Wallis test. Kruskal-Wallis one-way analysis of variance by ranks is an extension of the Mann-Whitney U test and is used to verify the hypothesis concerning the irrelevance of differences between medians of the studied variable in several populations, assuming that the variable distributions are close. The obtained results were considered statistically significant, with the significance level *p* < 0.05. Statistically significant differences or dependencies are marked in bold.

## Results

### Characteristics of the study group

The age of the women included in the study ranged from 45 to 65 (M = 57.07; DS = 4.74). Over half of the respondents lived in towns and cities (304; 59.6%) and 206 lived in rural areas (40.4%). The vast majority of the respondents completed secondary education (215; 42.2%). There were 170 women with higher education (33.3%), 81 completed basic vocational education (15.9%) and 44 had only primary education (8.6%).The vast majority of the respondents were married (380, 74.5%), 63 were widowed (12.4%), 35 women were single (6.9%), and 32 women (6.3%) were divorced.

The menopause of respondents started between ages 40 and 60 (M = 50.96, SD = 3.42, Q1 = 49, Q3 = 53). At the time of the study 54 women (10.6%) were on hormone replacement therapy (HRT). Out of these 54 respondents, 44 (81.5%) had been on this therapy for less than 5 years and the remaining 10 (18.5%) for over 5 years. 297 (58.2%) participants indicated that at the time of the study they were undergoing continuous treatment for chronic diseases. In order to ensure that the study group does not include respondents heavily affected by chronic diseases, the so-called outlier observations were rejected Then, statistical analysis was performed to check the differences between health behaviours of the respondents with and without chronic diseases (health behaviours were measured with the IHB questionnaire). There was no statistically significant difference in health behaviours between these groups of respondents (Z = -1.902; *p* = 0.057).

### Health self-assessment by postmenopausal women and their definition of health

More than a half of the respondents (298, 58.4%) rated their health as good; 172 (33.7%) as average; 21 (4.1%) as bad and 19 (3.7%) as very good.

In the view of the respondents health was primarily synonymous with a feature, because the following three associations were given the highest priority when defining health: to be healthy means ‘have all body parts functioning well’ (M = 1.82), ‘do not experience any physical problems’ (M = 1.43) and ‘not be sick, only occasionally suffer from flu, cold or indigestion’ (M = 1.30). Detailed results are presented in Table 1.

**Table 1:** Mean weights of items regarding own health in the examined group

Items	M	SD	Health definition
To be healthy means to me to have all body parts functioning well	1.82	2.06	Feature
To be healthy means to me to be happy most of the time	1.44	1.94	Condition
To be healthy means to me that I do not experience any physical problems	1.43	1.83	Feature
To be healthy means to me that I am not sick, I suffer from flu, cold or indigestion only occasionally	1.30	1.84	Feature
To be healthy means to me that I feel well and happy	0.96	1.58	Condition
To be healthy means to me that I have good social relations with other people	0.89	1.58	Process
To be healthy means to me that I am able to enjoy life	0.86	1.54	Condition
To be healthy means to me that I accept myself and I am aware of my abilities and shortcomings	0.73	1.47	Goal
To be healthy means to me that I am able to solve my problems	0.65	1.35	Process
To be healthy means to me that I have a proper diet	0.55	1.26	Result
To be healthy means to me that I am able to work without tension or stress	0.47	1.18	Process
To be healthy means to me that I need to take medications only occasionally	0.42	1.02	Feature
To be healthy means to me that I need to make doctor’s appointments hardly ever	0.41	1.08	Feature
To be healthy means to me that I devote enough time to relax and sleep	0.38	1.04	Result
To be healthy means to me that I am able to adapt well to changes in my life	0.35	1.03	Process
To be healthy means to me that my body weight is correct	0.34	0.94	Result
To be healthy means to me that I have a good mood	0.34	0.91	Feature
To be healthy means to me that I do not smoke	0.28	1.02	Result
To be healthy means to that I have a job and diverse interests	0.26	0.88	Result
To be healthy means to me to have healthy eyes, hair and skin	0.21	0.79	Feature
To be healthy means to me that I am responsible	0.21	0.85	Goal
To be healthy means to me that I can control my feeling and desires	0.10	0.55	Process
To be healthy means to me that I will live to my old age	0.08	0.47	Goal
To be healthy means to me that I drink alcohol only occasionally or never	0.07	0.48	Result

*M* mean/ average, *SD* standard deviation

Health self-assessment of the respondents was significantly linked with the importance assigned to the following health-related items: ‘have a proper diet’ (*p* = 0.007), ‘need to take medications only occasionally’ (*p* = 0.020), ‘not be sick and suffer from flu, cold or indigestion only occasionally’ (*p* = 0.002), ‘be responsible’ (*p* = 0.026), ‘accept yourself and be aware of your abilities and shortcomings’ (*p* = 0.049); ‘hardly ever need to make doctor’s appointments’ (*p* = 0.010). Detailed results are presented in Table 2.

**Table 2:** The mean significance of items and self-assessment of health in the study group

‘to be healthy means to me …. ‘	Self-assessment of health (mean rank)				Significance Kruskal-Wallis H test	
	Very good	Good	Average	Bad	H	p
To live to my old age	0.00	0.12	0.03	0.00	4.520	0.210
To feel well and happy most of the time	2.05	1.35	1.52	1.48	4.037	0.257
To have good social relations with other people	0.95	0.84	0.97	0.81	2.785	0.426
To be able to solve your own problem	1.00	0.66	0.61	0.57	2.150	0.542
To have a proper diet	0.32	0.65	0.48	0.00	**12.214**	**0.007**
To devote enough time to relax and sleep	0.32	0.38	0.45	0.00	4.382	0.223
To drink alcohol only occasionally or never	0.00	0.08	0.08	0.00	1.668	0.644
Not to smoke	0.00	0.32	0.25	0.24	2.265	0.519
To have correct body weight	0.26	0.30	0.44	0.24	0.921	0.820
To need to take medications only occasionally	0.32	0.33	0.52	0.86	**9.819**	**0.020**
To have a good mood	0.21	0.33	0.38	0.33	0.653	0.884
Not to feel any physical problems	0.68	1.39	1.50	2.10	7.626	0.054
To be able to work without tension or stress	0.16	0.55	0.40	0.19	7.288	0.063
Not to be sick and suffer from flu, cold or indigestion only occasionally	1.74	1.17	1.30	2.71	**14.971**	**0.002**
To have healthy eyes, hair and skin	0.16	0.18	0.30	0.00	2.700	0.440
To be able to adapt to changes in your life	0.26	0.38	0.35	0.10	1.323	0.724
To be able to enjoy life	1.68	0.89	0.78	0.33	4.019	0.259
To be responsible	0.42	0.29	0.05	0.14	**9.262**	**0.026**
To be able to control my emotions and desires	0.00	0.11	0.09	0.00	4.291	0.232
To have all body parts functioning well	1.68	1.75	1.92	2.10	1.411	0.703
To accept yourself, to be aware of your abilities and shortcomings	1.00	0.84	0.57	0.29	**7.840**	**0.049**
To have a job and diverse interests	0.53	0.32	0.16	0.10	6.778	0.080
To feel well	1.16	0.97	0.90	1.19	1.294	0.731
To need to make doctor’s appointments almost never	0.11	0.33	0.55	0.71	**11.378**	**0.010**

### Health behaviours of postmenopausal women

The range of raw scores obtained by the respondents who completed the IHB questionnaire ranged from 44 to 116 (M = 86.18; SD = 13.07). On the basis of standardized values, the result corresponded with the 5–6 sten scores and it was interpreted as average [25]. Analysis of all types of health behaviours showed that 127 (24.9%) of the respondents obtained low results in health-related behaviours (1–4 sten scores), 200 (39.2%) women obtained average results (5–6 sten scores), and 183(35.9%) respondents showed high scores (7–10 sten scores).

Participants gave the highest score to prophylactic behaviours (PBs) (M = 3.74; SD = 0.70). Next were a positive mental attitude (PMA) (M = 3.60; SD = 0.70), proper eating habits (PEH) (M = 3.55; SD = 0.73), and health activities (HA) (M = 3.47; SD = 0.75).

For the purpose of statistical analysis, participants were divided into two age groups: women under 55 years of age (204; 40%) and women over 55 years of age (306; 60%). There was a significant correlation between health self-assessment and the general indicator of health-related behaviours in both age groups of respondents. General indicator of health-related behaviours was higher in women who rated their health as very good (under 55 years of age: M = 98.00; *p* = 0.007, and over 55 years of age: M = 94.44; *p* = 0.019). Similarly, women in both age groups who assessed their health as very good have achieved statistically higher scores for HA (under 55 years of age: M = 4.17; *p* = 0.009 and for women over 55 years of age: M = 3.98; *p* < 0.001). In addition, women under 55 years of age who assessed their health as very good have achieved higher scores for PMA (M = 4.43; *p* = 0.001). Detailed results are presented in Table 3.

**Table 3:** Health behaviours and self-assessment of health in postmenopausal women

Variables			Self-assessment of health				(Kruskal-Wallis H test)
			Very good *n* = 19; 3.7%	Good *n* = 298; 58.4%	Average *n* = 172; 33.7%	Bad *n* = 21; 4.1%	
HBI	**< 55**	M	98.00	84.95	83.44	85.33	**H = 12.11** ***p*** **= 0.007**
		SD	8.79	12.40	11.15	8.08	
HBI	**> = 55**	M	94.44	86.43	87.76	80.56	**H = 9.98** ***p*** **= 0.019**
		SD	11.52	12.68	15.06	10.61	
PEH	**< 55**	M	3.57	3.54	3.40	3.39	H = 2.02 *p* = 0.568
		SD	0.47	0.68	0.73	0.59	
PEH	**> = 55**	M	3.89	3.57	3.60	3.33	H = 3.42 *p* = 0.331
		SD	0.44	0.73	0.80	0.74	
PBs	**< 55**	M	4.17	3.65	3.78	4.00	H = 6.52 *p* = 0.089
		SD	0.32	0.69	0.69	0.67	
PBs	**> = 55**	M	3.91	3.75	3.74	3.78	H = 0.24 *p* = 0.971
		SD	0.71	0.70	0.76	0.51	
PMA	**< 55**	M	4.43	3.55	3.50	2.94	**H = 17.18** ***p*** **= 0.001**
		SD	0.52	0.60	0.62	0.82	
PMA	**> = 55**	M	3.96	3.69	3.58	3.22	H = 5.27 *p* = 0.153
		SD	0.73	0.68	0.78	0.98	
HA	**< 55**	M	4.17	3.41	3.29	3.89	**H = 11.62** ***p*** **= 0.009**
		SD	0.82	0.62	0.77	0.51	
HA	**> = 55**	M	3.98	3.40	3.66	3.09	**H = 20.22**
		SD	0.54	0.72	0.85	0.59	***p*** **< 0.001**

*HBI* the general ratio of health behaviour inventory, *PEH* positive eating habits, *PBs* prophylactic behaviours, *PMA* positive mental attitude, *HA* health activities, *M* mean result, *SD* standard deviation

### Health behaviours and preventive examinations

The study indicated that 291 (57.1%) of the respondents underwent prophylactic gynaecological examinations on regular basis. Out of this group, 163 women reported for the gynaecological examination once a year (32.0%), 81(15.9%) - once in 2 years, and 47 (9.2%) had a prophylactic gynaecological examination performed every half a year. 159 (31.2%) respondents underwent such examinations sporadically, and 60 (11.7%) women had never had such examinations done. 58 (11.4%) women admitted that they see their gynaecologist only when some disturbing symptoms occur. 2(0.4%) respondents claimed they had never visited the gynaecologist.

The most common reason for reporting for a gynaecological examination in the previous year was a general check-up (282; 55.3%). Less frequently quoted reasons were some alarming symptoms, which respondents wanted to consult or continuation of the treatment – 190 (37.3%) and 32 (6.3%) respondents respectively. 6 (1.2%) women did not see a gynaecologist in the previous year.

The study revealed that 369 (72.4%) women performed breast self-examinations. 51 (13.8%) respondents did it on a regular basis once a month, and 19 (5.1%) claimed they self-examined their breasts every 2 months. The rest (299; 58.6%) said they did it occasionally. 17 (3.3%) of the respondents declared they had had breast ultrasound performed while 493 (96.7%) women admitted that they had never had it done. A regular mammography screening was declared by 369 (72.4%) respondents, and 141 (27.6%) had never reported for this examination. Similar results were obtained in the case of cervical smear test − 354 women had it done regularly (69.4%), whereas 156 respondents had it done occasionally or not at all (30.6%). 175 women (34.3%) underwent other (non-gynaecological) prophylactic examinations, whereas 335 (65.7%) denied having had any other (non-gynaecological) prophylactic examinations performed.

Women in both age groups whose general indicator of health-related behaviours was higher regularly performed prophylactic gynaecological examinations (M = 87.03; *p* = 0.016 for women under 55 years of age, and M = 90.45; *p* < 0.001 for women over 55 years of age). Also those who obtained higher results in PEH (M = 3.61; *p* = 0.001 for women under 55 years of age, and M = 3.76; *p* < 0.001 for women over 55 years of age), higher results in PBs (M = 3.85; *p* = 0.001 for women under 55 years of age, and M = 3.95; *p* < 0.001 for women over 55 years of age), and higher results in HA (M = 3.50; *p* = 0.032 for women under 55 years of age, and M = 3.62; *p* = 0.009 for women over 55 years of age). Detailed results are presented in Table 4.

**Table 4:** Health behaviours of women and the performance of prophylactic gynaecological examinations

Variables			Performance of prophylactic gynaecological examinations			(Kruskal-Wallis H test)
			Regular *n* = 291; 57.1%	Irregular *n* = 159; 31.2%	Never *n* = 60; 11.7%	
HBI	**< 55**	M	87.03	82.23	80.80	**H = 8.30**
		SD	11.11	13.48	13.41	***p*** **= 0.016**
HBI	**> = 55**	M	90.45	82.86	83.10	**H = 21.69**
		SD	13.21	13.44	11.91	***p*** **< 0.001**
PEH	**< 55**	M	3.61	3.42	3.03	**H = 13.76**
		SD	0.63	0.73	0.67	***p*** **= 0.001**
PEH	**> = 55**	M	3.76	3.38	3.38	**H = 19.25**
		SD	0.73	0.75	0.66	***p*** **< 0.001**
PBs	**< 55**	M	3.85	3.41	3.65	**H = 13.32**
		SD	0.58	0.68	1.02	***p*** **= 0.001**
PBs	**> = 55**	M	3.95	3.56	3.47	**H = 23.98**
		SD	0.63	0.75	0.73	***p*** **< 0.001**
PMA	**< 55**	M	3.57	3.63	3.41	H = 1.63
		SD	0.64	0.65	0.55	*p* = 0.443
PMA	**> = 55**	M	3.72	3.51	3.58	H = 5.36
		SD	0.73	0.77	0.71	*p* = 0.069
HA	**< 55**	M	3.50	3.25	3.39	**H = 6.89**
		SD	0.69	0.69	0.65	***p*** **= 0.032**
HA	**> = 55**	M	3.62	3.35	3.42	**H = 9.41**
		SD	0.84	0.69	0.69	***p*** **= 0.009**

*HBI* the general ratio of health behaviour inventory, *PEH* positive eating habits, *PBs* prophylactic behaviours, *PMA* positive mental attitude, *HA* health activities, *M* mean result, *SD* standard deviation

Women under 55 years of age who obtained statistically higher results in health behaviours regarding PEH (M = 3.56; *p* = 0.034) and PMA (M = 3.63; *p* = 0.034) regularly performed breast self-examinations. Also those over 55 years of age who obtained statistically higher results in health behaviours regarding PBs (M = 3.81; *p* = 0.025), and PMA (M = 3.68; *p* = 0.042). Detailed results are presented in Table 5.

**Table 5:** Health behaviours of postmenopausal women and the performance of breast self-examination

Variables			Breast self-examination		(Mann-Whitney U test)
			Regular *n* = 369; 72.4%	Irregular *n* = 141; 27.6%	
HBI	**< 55**	M	85.85	83.08	Z = -1,64
		SD	11.50	14.12	*p* = 0.100
HBI	**> = 55**	M	87.75	84.75	Z = -1,34
		SD	13.44	13.88	*p* = 0.180
PEH	**< 55**	M	3.56	3.33	**Z = -2.12**
		SD	0.63	0.82	***p*** **= 0.034**
PEH	**> = 55**	M	3.61	3.51	Z = -0.81
		SD	0.73	0.79	*p* = 0.419
PBs	**< 55**	M	3.76	3.60	Z = -1.53
		SD	0.64	0.80	*p* = 0.127
PBs	**> = 55**	M	3.81	3.61	**Z = -2.24**
		SD	0.70	0.74	***p*** **= 0.025**
PMA	**< 55**	M	3.63	3.38	**Z = -2.11**
		SD	0.60	0.70	***p*** **= 0.034**
PMA	**> = 55**	M	3.68	3.50	**Z = -2.04**
		SD	0.73	0.76	***P*** **= 0.042**
HA	**< 55**	M	3.39	3.54	Z = -1.43
		SD	0.68	0.73	*p* = 0.152
HA	**> = 55**	M	3.50	3.51	Z = -0.14
		SD	0.76	0.82	*p* = 0.891

*HBI* the general ratio of health behaviour inventory, *PEH* positive eating habits, *PBs* prophylactic behaviours, *PMA* positive mental attitude, *HA* health activities, *M* mean result, *SD* standard deviation

Women over 55 years of age whose general indicator of health-related behaviours was statistically higher (M = 88.22; *p* = 0.005) and who obtained higher results in health behaviours regarding PBs (M = 3.86; *p* < 0.001) more often had regular mammography screening. And in the group of younger women, under 55 years of age, the regularity of this examination was more frequent among these respondents who obtained statistically higher results in PEH (M = 3.58; *p* = 0.037). Detailed results are presented in Table 6.

**Table 6:** Health behaviours of postmenopausal women and the use of mammography

Variables			Mammography examination		(Mann-Whitney U test)
			Regular *n* = 369; 72.4%	Irregular *n* = 141; 27.6%	
HBI	**< 55**	M	85.63	84.35	Z = -0.59
		SD	11.18	13.99	*p* = 0.557
HBI	**> = 55**	M	88.22	82.33	**Z = -2.80**
		SD	13.24	13.97	***p*** **= 0.005**
PEH	**< 55**	M	3.58	3.35	**Z = -2.09**
		SD	0.58	0.83	***p*** **= 0.037**
PEH	**> = 55**	M	3.63	3.41	Z = -1.75
		SD	0.74	0.78	*p* = 0.081
PBs	**< 55**	M	3.75	3.66	Z = -0.65
		SD	0.62	0.80	*p* = 0.516
PBs	**> = 55**	M	3.86	3.38	**Z = -4.68**
		SD	0.66	0.76	***p*** **< 0.001**
PMA	**< 55**	M	3.58	3.54	Z = -0.53
		SD	0.64	0.63	*p* = 0.594
PMA	**> = 55**	M	3.66	3.54	Z = -1.14
		SD	0.72	0.82	*p* = 0.253
HA	**< 55**	M	3.38	3,51	Z = -1.17
		SD	0.71	0.66	*p* = 0.242
HA	**> = 55**	M	3.55	3.34	Z = -1.84
		SD	0.77	0.79	*p* = 0.066

*HBI* the general ratio of health behaviour inventory, *PEH* positive eating habits, *PBs* prophylactic behaviours, *PMA* positive mental attitude, *HA* health activities, *M* mean result, *SD* standard deviation

## Discussion

### Study group

The study group was differentiated by several sociodemographic factors, most importantly age of the participants, as it ranged from 45 to 65 years. Obviously, it was due to the inclusion criteria adopted in this study, which referred not to the chronological age, but to the time which lapsed since the last menstrual period, and also due to quite large time span (between 2 and 10 years of the menopause) accepted for the study. Participants of this study went through menopause between the age of 40 and 60. This is consistent with population studies concerning Poland [26] and other highly developed countries [27, 28].

The diversity concerning the place of living provides, according to some authors, the possibility of identifying beneficial as well as adverse aspects of living in urban and rural areas [29, 30]. It is worth emphasizing that the differences between these areas are becoming less and less noticeable. In this study the number rural residents was significant (40.4%). The vast majority of the respondents (75.5%) declared to have completed secondary education. Study groups in similar studies conducted by other authors also comprised women with similar educational background [31], however some other authors noted a higher percentage of participants with basic vocational education [32]. According to broad population studies, middle aged women are characterized by a lower level of education than the study group in the presented material [33].

Hormone replacement therapy was used by 10.6% of respondents at the time of the study. In the light of reports from literature, this percentage should be considered as relatively low, because, as some authors claim, climacteric syndrome symptoms appear in 75% of perimenopausal women, and 25% of them require treatment [34]. Hormone replacement therapy is effective in relieving menopausal symptoms, i.e. hot flushes, night sweats, dyspareunia, sexual dysfunction and insomnia, as well as in the prevention of osteoporosis [34, 35]. However, there are some contraindications to the use of this therapy [36].

### Health self-assessment by postmenopausal women and their definition of health

In the presented material, an attempt was made to define the concept of ‘being healthy’ as understood by the postmenopausal women. The obtained results proved that the respondents perceived health primarily as a feature (to have all parts of the body functioning well and not to feel any physical discomfort) and/or condition (to experience happiness most of the time). These results are similar to the results obtained in other studies which were carried out on groups of elderly people [37, 38]. There are also studies showing that health is perceived as a feature also by younger people, i.e. over 40 years of age [39], and by chronic patients [40].

The analysis of the presented results showed that the definition of health was related to the self-assessment of health. The study proved that women with low self-assessment of their health more frequently understood health instrumentally. Interestingly, these participants selected the statements which defined health as a feature of a body (to take medications only occasionally, not be sick or only suffer from flu, cold or indigestion, not need to make appointments with a doctor and/or hardly ever go to the doctor). On the other hand, those who assessed their own health better were more likely to choose claims that corresponded to the definition values of the result (to eat properly) or purpose (to accept oneself, to know your capabilities and deficiencies). In his study, Juczyński noted that low self-assessment of health was associated with attaching greater importance to the physical criteria of health [25]. Moreover, there are differences in the way health is understood in the case of loss of health or the occurrence of chronic illness [3]. Thus, health self-assessment is gaining popularity in the field of epidemiological research where is employed to assess the health condition of entire populations [41]. Additionally, some authors notice a correlation between health self-assessment and the results of laboratory tests and the prevalence of various civilization diseases [42]. In this study self-assessment of health proved to be surprisingly high. It was rated as good by more than half of the respondents, even though they were undergoingcontinuous treatment for various chronic diseases. The literature review shows that hypertension, coronary heart disease and atherosclerosis are the main medical problems in the postmenopausal period [43, 44].

### Health behaviours of postmenopausal women

The overall rate of health behaviours of the women in the studied group was average (M = 86.18, SD = 13.08). The results proved to be comparable with the normalized results of Juczyński (M = 85.98, SD = 12.70) who observed that the postmenopausal women exhibit more behaviours that have a positive effect on health than younger women. Juczyński claims that the only exception to this observation are the younger women who are affected by some chronic diseases [25]. Recent years indicate a fairly constant tendency among older women to improve their health behaviours. According to some authors, seniors may even show above-average results [45]. However, the study by Kurowska and Kierzenkowska [32] shows the opposite trend – women over 60 have worse results in the area of pro-health behaviours. The results of the present study indicate that prevailing pro-health activities encompass prophylactic behaviours. Postmenopausal women should be under a regular care of a gynaecological clinic, just like younger women, and the frequency and type of appointments should be agreed individually, depending on the needs [46]. Nevertheless, gynaecological check-ups should take place at least once a year [14].

Our study revealed that slightly more than a half of the respondents regularly had a prophylactic gynaecological examination, and only 32.0% of them did so in line with the above-mentioned recommendations. In addition, the study confirmed that some women (11.7%) had never had a prophylactic gynaecological examination performed. It is probable they would never see a doctor without a serious reason, which could be considered a risky behaviour once they reached the postmenopausal period. According to literature, the frequency of women reporting for gynaecological examinations decreases with age, and women between 41 and 60 report to the gynaecologist less frequently than every 20 months [47]. This situation should be considered as both worrying and requiring improvement. This study shows that in many cases (37.3%) the only reason for making an appointment with a gynaecologist was the appearance of disturbing symptoms. Such appointments do not have a prophylactic character. Some authors claim that such appointments are perceived by many women as a compulsion or an indispensable duty. They feel exempt from this duty if there are no disturbing symptoms [14]. Sometimes even when symptoms do show up (including the climacteric syndrome), it does not increase the regularity of gynaecological check-ups [48]. Breast self-examination is the first step in the secondary prophylactics of breast cancer. It is a simple, inexpensive, fast and non-invasive examination and all women should be encouraged to be more actively responsible for their own health [49]. It is the self-examination of breast that increases the number of early detections of breast cancers and therefore women should be encouraged to perform this self-check on a regular basis [14]. Our study indicated that 72.4% of women perform breast self-examination, although only a few (13.8%) did it regularly on a monthly basis. Similar trend was observed by other researchers [50]. One of the possible manifestations of women’s concerns for their own health is taking advantage of free prophylactic examinations. According to the National Health Fund (NFZ), in 2015 only one in five women took part in the Population-Based Breast Cancer Early Detection Program, and in 2018 nearly two times more women participated. The Population-Based Cervical Cancer Screening Program attracted even fewer women-9.34 and 17.89%, respectively [51]. Our study indicated that 72.4% of the respondents declared undergoing regular mammography examinations and 69.4% confirmed they undergo regular smear tests of the cervix. However, it is not known to what extent this was a participation in a population-based screening programme. Perhaps some of them decided to undergo these examinations on their own initiative, i.e. without an invitation. What is more, some women sign up for test in private clinics. Anyway, the attendance rate is still unsatisfactory [52]. The reasons for such low attendance rate may be numerous and include a lack of faith in their effectiveness, ignoring the problem of cancer, the fear of pain and nudity associated with the examination, as well as fear of detecting the disease [53].

As regards health behaviours concerning positive mental attitude (PMA), the following categories were taken into account: avoidance of upsetting and depressing situations, avoidance of excessive emotions and tensions, and social life. The analysed material showed quite high psychometric properties of this factor (M = 3.60; SD = 0.70), which can be considered beneficial for the mental health of postmenopausal women. This is good news, as in this age group the incidence of various mental disorders, especially depression and anxiety, is generally on the increase [9]. These women, when compared to younger women, feel more negative emotions, such as anxiety, sadness and exhaustion [49].

Proper eating habits (PEH) are the third important health criterion and a number of factors were taken into account including the frequency of consumption of fruit, vegetables and wholegrain bread, and decrease in the consumption of animal fats, sugar, salt and heavily salted foods. The literature emphasizes the importance of following the principles of healthy nutrition and proper diet in the prophylaxis of diseases typical for the postmenopausal period (metabolic syndrome, ischemic heart disease, diabetes, malignant tumors, osteoporosis and depressive disorders) [14, 54].

It is worth noting that Juczyński [25] presented an identical distribution of results for all categories of health behaviours in his study. It is undeniable, however, that the results obtained by the authors of this study as well as the results obtained by other authors show that women are not sufficiently concerned about their own health. The average results which were obtained in reference to health-related behaviours cannot be considered satisfactory, due to the fact that women in this period are more susceptible to various psychophysical disorders [9, 14, 55, 56].

Choosing pro-health behaviours is usually characteristic of people who are satisfied with their health [57]. In the presented material higher self-assessment of health was significantly associated with a higher general indicator of health-related behaviours. In addition, in both age groups women who regularly performed prophylactic gynaecological examinations obtained higher score of the general indicator of health-related behaviours, proper eating habits (PEH), prophylactic behaviours (PBs) and health activities (HA). Moreover, women over 55 years of age, who achieved higher scores in prophylactic behaviours (PBs) had mammography screening and preformed self-examination of breasts more regularly.

The obtained results concerning the concept of health, health self-assessment and the type of health behaviours undertaken by postmenopausal women may be further used in broadly defined health promotion programs, including new prophylactic programs. Most of these programs are aimed at convincing women that the proposed health-related behaviours will not only improve their lives but also they will be beneficial for their families and society. However, the programs need to be constantly improved and adapted to changing needs.

### Limitation of the study

This study has several important limitations that may affect the obtained results. First and foremost, the selection of the study sample using convenience sampling methodology. Next, the broad age range of women included in the study. Therefore, for the purpose of statistical analysis, the study group was divided into two age groups. This way it was possible to show in more detail any possible differences in health behaviours and in the undertaken prophylactic activities. Another limitation is connected with the inclusion of women who had reported that they were undergoing continuous treatment for chronic diseases at the time of the study, which could have modified their health behaviours. However, due to the age of the participants, it is difficult, if at all possible, to include only women without any ongoing health problems. Therefore, to minimize this limitation, a statistical analysis was performed to check any potential differences in health behaviours presented by women in these two groups (with and without chronic diseases). The analysis showed that there is no statistically significant difference between these women in terms of health behaviours. It has to be noted that the claim of an undergoing treatment for a chronic disease was made subjectively by the participants. Their health history was not examined to objectify the results, neither were their former health behaviours investigated. Therefore, it was impossible to compare and analyse any changes, which could have occurred in this regard. It would be advisable to carry out such analyses in the future using a mix-method methodology, supplementing the collected material with qualitative research, which would allow for a more in-depth analysis of the issue.

## Conclusions

The presented results showed that women after menopause treat health mainly as a as a feature of their body and condition specific for this period of life. Therefore, it is very important to engage medical personnel, mainly nurses and midwives, in making this group of women aware that they have an impact on the biological, psychological and social sphere of their own health. Furthermore, after the analysis of postmenopausal women’s health behaviours and their perception of health, the areas have been identified that require the focus of medical personnel in regard to health promotion and prophylaxis. The average general indicator of health-related behaviours is positive for this group of women as it shows that they care about their health, especially in terms of prophylaxis. This is also reflected in the fact that the majority of postmenopausal women declare regular breast self-examination and mammography screening.

The postmenopausal period is characterized by a number of biological symptoms, as well as changes in mental and social functioning, with a tendency to depressive disorders. Among the respondents this trend was expressed by the average behavioural indicator in the area of positive mental attitudes and it was not related to the age of respondents. This suggests the need to pay particular attention to mental health hygiene in this group of women. Care providers for this group should instruct women on how to cope with difficult situations and give vent to tensions and, in some situations, enable access to mental health professionals. A properly balanced diet, regular meals and physical activity have an impact on the health of women of all ages, but are especially important when the metabolism changes in the postmenopausal period. Educational activities aimed at encouraging women to change their lifestyle, with a particular attention to optimal nutrition and active forms of recreation, would be a very good solution.

The results of this study suggest the need for creating an attitude in postmenopausal women of active involvement in their own healthcare. This would contribute to the reduction of health problems in this group and improve the quality of life.

## Supplementary information

**Additional file 1.**

## Data Availability

Data are available by contacting the corresponding author.

## Abbreviations

WHO: World Health Organization
STROBE: Strengthening the Reporting of Observational studies in Epidemiology studies
LHC: The List of Health Criteria
IHB: Inventory of Health Behaviour
NFZ: The National Health Fund
PMA: Positive mental attitude
PEH: Proper eating habits
PBs: Prophylactic behaviours
HA: Health activities
