# Supplementary material for: Perception of health, health behaviours and the use of prophylactic examinations in postmenopausal women
Source: BMC Womens Health. 2020 Apr 9;20:71. doi: 10.1186/s12905-020-00931-9 (PMC7155283; doi:10.1186/s12905-020-00931-9)
Supplement: Supplementary file 1 — Additional file 1. [file 12905_2020_931_MOESM1_ESM.docx]

**Questionnaire to collect sociodemographic data**

Dear Madam!
I kindly ask you to take part in the study on health behaviors of postmenopausal women. The data will be used for a scientific study aimed at improving the quality of care for this group of people. Research is voluntary and anonymous.

Thank you very much for answering questions

- How old are you? ......
- Could you write what is your body weigh? ………..
- Could you write what is your height? ………..
- What is your education?
   primary  junior high school  basic professional/vocational
   secondary education  bachelor’s degree  higher education (master’s degree and higher)
- Place of residence:
   city

 village

- Marital status:
   single
   married

 divorcee
 widow

- At what age did you have your last menstrual period? ........
- Do you currently use hormone replacement therapy?
   yes - since what time? ...............
   no - why?
   I did not agree to such therapy
   I'm afraid to take hormonal preparations
   I don't know anything about this therapy
   for financial reasons
   for another reason - which one? ........................................... .
- What type of hormone replacement therapy did you use or do you currently use?
   Slices

 tablets

 injections
 gels

 vaginal globules

 implants
 I do not use

- Are you currently treated for chronic diseases?
   yes - which ones? ............................................. .................
   not
- How would you assess your health?
   very good

 good
 sufficient

 bad

- How often do you visit a gynecologist for preventive examinations?
   regularly once every six months
   regularly once a year
   regularly once every two years
   less than once every two years
   I do it irregularly
   I report only when symptoms that disturb me appear
   I've never been to a gynecologist
- Why did you go to a gynecologist last year?
   wanting to check your health
   worrying symptoms appeared
   continuation of treatment
   N/A
- Do you have the habit of regularly reporting for preventive examinations, such as:
  mammography:
   yes  no
  Cytological examination of the cervix:
   yes  no
- Did you receive a personal invitation to participate in preventive examinations, such as:
  mammography:
   yes  no
  Cytological examination of the cervix:
   yes  no
- Do you perform breast self-examination?
   yes - how often?
   regularly every month
   regularly every 2 months
   when I remember
   no - why?
   I don’t know how
   I don't want to do this
   I'm afraid to detect a change
   I forget about it
   for another reason - which one? ........................................... ...
- Do you apply for other preventive examinations?
   yes - what? ............................................. ......................
   not
